# Resveratrol inhibits age-dependent spontaneous tumorigenesis by SIRT1-mediated post-translational modulations in the annual fish *Nothobranchius guentheri*

**DOI:** 10.18632/oncotarget.19268

**Published:** 2017-07-15

**Authors:** Tingting Liu, Long Ma, Zhaodi Zheng, Fenglin Li, Shan Liu, Yingbo Xie, Guorong Li

**Affiliations:** ^1^ Shandong Provincial Key Laboratory of Animal Resistance Biology, School of Life Sciences, Shandong Normal University, Jinan, China

**Keywords:** spontaneous tumorigenesis, resveratrol, SIRT1, post-translational modulations, annual fish

## Abstract

Resveratrol, SIRT1 activator, inhibits carcinogenesis predominantly performed in transgenic animal models, orthotopic cancers of nude mice or different cancer cell lines, but its effects during process of spontaneous tumors using vertebrate models remain untested. Spontaneous liver neoplasm is an age-related disease and is inhibited by resveratrol in the annual fish *Nothobranchius guentheri*, which indicates that the fish can act as an excellent model to study spontaneous tumorigenesis. Totally, 175 fish were fed with resveratrol and another 175 fish for controls. Treated fish were fed with resveratrol (25 μg/fish/day) from sexual maturity (4-month-old) until they were sacrificed at 6-, 9- and 12-month-old. Immunoblot, immunohistochemistry and co-immunoprecipitation were employed to investigate the underlying mechanisms that resveratrol inhibited age-dependent spontaneous tumorigenesis in the fish. Results showed that resveratrol increased protein level of SIRT1 and alleviated age-associated tumorigenesis in liver. With SIRT1 up-regulation, resveratrol reduced proliferation by deacetylating K-Ras and inactivating K-Ras/PI3K/AKT pathway; and promoted apoptosis through deacetylation and dephosphorylation of FoxOs, up-regulation of DLC1 and interaction between SIRT1 and DLC1, and dephosphorylation of DLC1 in spontaneous neoplasms. We established a novel short-lived fish model for understanding the molecular mechanisms of drugs on age-dependent spontaneous tumorigenesis.

## INTRODUCTION

Animals are reliable tools for modeling human diseases. Rats, mice and zebrafish have been used to understand the pathogenesis of cancers *in vivo* systems. In diethylnitrosamine-induced hepatocellular carcinoma of rats, curcumin ameliorates carcinoma by down-regulation of AKT and up-regulation of caspase-3 [1], and resveratrol prevents hepatocarcinogenesis by activating proapoptotic proteins [2]. In nude mice, resveratrol inhibits orthotopic pancreatic tumors by PI3K/AKT pathway [3], capsaicin suppresses pancreatic tumor xenografts by JNK/FoxO/Bim cascade [4], and resveratrol induces apoptosis of prostate cancer xenografts by inhibition of phosphorylation of FoxOs [5]. Transgenic zebrafish are also employed to explore mechanisms of tumorigenesis, as Tol2 and CRISPR/Cas9 system approaches have been successfully applied. Activating mutations in *K-Ras* oncogene drives robust liver tumorigenesis by PI3K/AKT signal; Erk and Stat5 are involved in formation of hepatocellular carcinoma in Tet-on *xmrk* transgenic zebrafish [6–8]. But these studies are predominantly conducted in diethylnitrosamine-initiated carcinoma of rats, orthotopic cancers of nude mice or transgenic zebrafish due to their long lifespan. The molecular mechanisms of spontaneous tumors need to be further clarified.

Compared to rats, mice and zebrafish, annual fishes of the genus *Nothobranchius* with short lifespan and accelerated growth are easy to raise and reproduce in a large scale. *N. furzeri, N. rachovii, N. guentheri* and *N. korthausae* (with mean lifespan of 3-6.5, 8.5, 12 and 16.5 months), are usually used to study the process of aging. With age, activities of antioxidant enzymes show a downward trend in *N. rachovii* and *N. guentheri* [9, 10], and cognitive performances show an age-dependent decrease in *N. furzeri* [11]. Moreover, previous studies show that spontaneous neoplasms develop in old fish. Liver and kidney show marked tumorous growth with the degeneration of thymus in aged *N. guentheri* [12–14], incidences of liver and gonads neoplasms are higher at 28 weeks than that at 11 weeks in *N. furzeri* [15], and the number of fish with hepatic neplasms is larger at 61 weeks than that at 28 weeks in *N. korthausae* [16]. And Di Cicco identifies that *N. furzeri* acts as a model to analyze age-dependent spontaneous tumorigenesis [15]. However, underlying mechanisms of spontaneous neoplasms and if *N. guentheri* can be used for spontaneous neoplasms remain largely unknown.

Resveratrol, a polyphenol occurs naturally in different plants, is mostly abundant in grapes and red wine. Resveratrol extends lifespan, alleviates oxidative stress and protects neurons in *N. guentheri* [10, 17, 18], attenuates fibrosis, inflammation and hepatic steatosis in mice [19, 20], and has therapeutic potential against myeloproliferative neoplasms [21]. Our previous results show that resveratrol extends lifespan and decreases oxidative stress of *N. guentheri*, which has the mean lifespan of 12 months [10, 18]. We further found that spontaneous liver neoplasm was observed in 9-month-old fish, and its incidence approached to 100% at 12-month-old. Resveratrol decreased age-dependent spontaneous tumorigenesis in the annual fish.

Based on these findings, we aimed to explore underlying mechanisms that resveratrol reduced spontaneous liver neoplasm in the fish. Here we reported that resveratrol decreased spontaneous tumorigenesis, and these phenotypes were associated with reduction of proliferation and promotion of apoptosis accompanying with SIRT1 up-regulation. Then the protein levels and post-translational modulations of K-Ras, FoxOs and DLC1 were detected to investigate the mechanisms that resveratrol inhibited age-dependent spontaneous tumorigenesis by SIRT1-mediated post-translational modulations in the annual fish.

## RESULTS

The mean lifespan of *N. guentheri* is 12-month-old, so we examined fish in three stages, 6-, 9- and 12-month-old, which represents young, middle-aged and old fish respectively.

### Resveratrol attenuated age-dependent spontaneous tumorigenesis in liver

**Figure 1:**
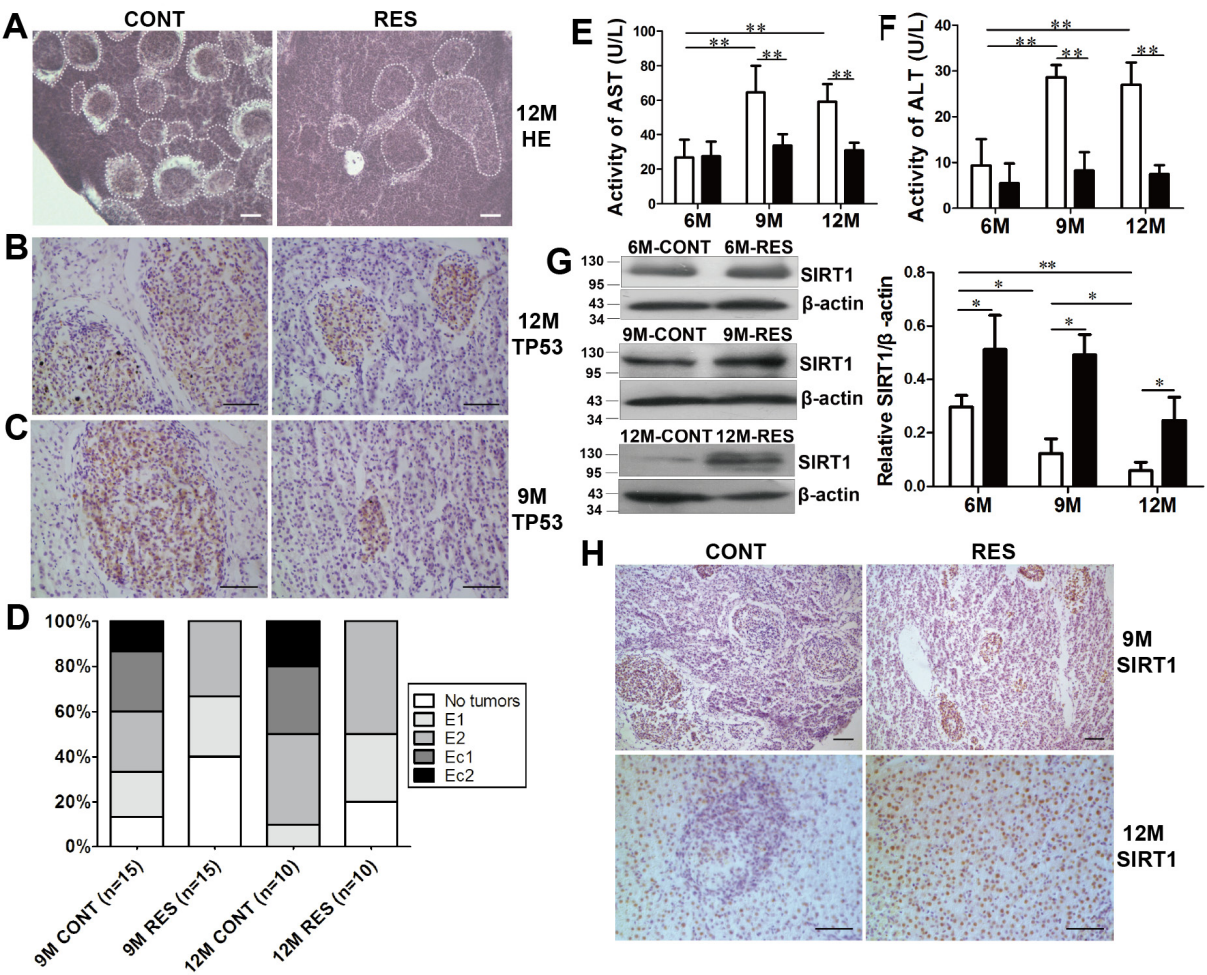
Resveratrol decreased age-dependent spontaneous tumorigenesis and increased protein level of SIRT1 in fish liver **(A)** Histological observations in liver at 12-month-old. Dotted areas indicated the spontaneous neoplasms. Scale bar = 30 mm. CONT: control group, RES: resveratrol group. **(B, C)** Positive immunohistochemical reaction to mutant anti-p53 antibody in hepatic neoplasms. Scale bar = 50 mm (n = 3 in each group). **(D)** Malignant degree of neoplasms in 9- and 12-month-old fish. **(E, F)** Activities of serum hepatic marker enzymes (AST and ALT) at three developmental stages (n = 6 in each group). White: CONT group; black: RES group. **(G, H)** SIRT1 expression was detected by immunoblot and immunohistochemical staining. Scale bar = 50 mm (n = 3 in each group). Data were presented as mean±SD. *p <0.05, **p <0.01.

Firstly, livers from middle-aged and old fish were bigger and relative liver weights were significantly higher when compared with young fish, and resveratrol decreased relative liver weights at the two stages by long-term feeding (Supplementary Figure 1). Next, histological analyses were performed to qualitatively examine the livers in old fish. The results showed that spontaneous liver neoplasms were developed clearly in 12-month-old fish, and resveratrol inhibited growth of neoplasms (Figure 1A). Immunoreactivity indicated that only foci of neoplasm nodules showed mutant p53 (TP53) positive signal, and these hepatic neoplasms are malignant tumors [15] (Figure 1B). Subsequently, livers from fish at 9- and 6-month-old were examined. Similar to old fish, spontaneous neoplasms were also found in 9-month-old fish, and resveratrol decreased mutant p53 staining (Figure 1C, Supplementary Figure 2). But there were no neoplasms both in control and resveratrol-fed fish at 6-month-old.

Spontaneous neoplasms incidence (of 30 fish at 9-month-old and 20 fish at 12-month-old) was calculated quantitatively. Neoplasm incidence showed an age-dependent increase and was decreased by resveratrol. As aging, the percentage of neoplasms was 86.67% (13/15) at 9-month-old and it approached to 100% (10/10) at 12-month-old. Resveratrol decreased percentage of spontaneous neoplasms both at 9-month-old (60%, 9/15) and 12-month-old (80%, 8/10) (Table 1). Control fish formed larger neoplasms, whereas fish fed with resveratrol formed smaller neoplasms. The number and size of spontaneous neoplasm nodules decreased in resveratrol-fed fish compared with control group (Table 2).

**Table 1:** Effects of resveratrol on spontaneous neoplasm at 6, 9- and 12-month-old

Groups	No. of fish with nodules/total fish	Neoplasm incidence (%)	Total no. of nodules	Average no. of nodules/nodule-bearing liver
6M CONT	0/10	0	0	0
6M RES	0/10	0	0	0
9M CONT	13/15	86.67%	72	5.54±2.88
9M RES	9/15	60%	31	3.44±2.19*
12M CONT	10/10	100%	90	9.00±5.91
12M RES	8/10	80%	27	3.38±1.41^#^

^*^P < 0.05: 9M RES compared with 9M CONT.

^#^P < 0.05: 12M RES compared with 12M CONT.

**Table 2:** Effects of resveratrol on neoplasm nodular size at 9- and 12-month-old

Groups	No. of fish with nodules/total fish	Total no. of nodules	>100 μm	75-100 μm	50-75 μm	<50 μm
9M CONT	13/15	72	9(12.5%)	25(34.72%)	31(43.06%)	7(9.72%)
9M RES	9/15	31	1(3.23%)	3(9.68%)	7(22.58%)	20(64.52%)
12M CONT	10/10	90	10 (11.11%)	29(32.22%)	27(30%)	24(26.67%)
12M RES	8/10	27	2(7.41%)	7(25.93%)	10(37.04%)	8(29.63%)

Spontaneous liver neoplasms at different levels of malignancy were defined according to scoring system used in *N. furzeri* [15]. The four levels of malignancy were single hepatoma or few small hepatomas (E1), several and well-differentiated hepatomas (E2), small and diffused hepatocellular carcinomas (Ec1) and diffused hepatocellular carcinomas (Ec2). Figure 1D showed that resveratrol decreased malignant degree of spontaneous neoplasms at 9- and 12-month-old.

### Resveratrol reduced activities of serum hepatic marker enzymes

Activities of serum hepatic marker enzymes are higher in diethylnitrosamine-induced hepatocellular carcinoma of rats [1, 22], so levels of aspartate aminotransferase (AST) and alanine transaminase (ALT) were measured at three different stages of the annual fish in with or without spontaneous neoplasms. Activities of AST and ALT at 9- and 12-month-old were much higher than that at 6-month-old in control fish, and resveratrol reduced their activities efficiently at the two stages (p<0.01) (Figure 1E, 1F). The spontaneous neoplasms developed in fish at 9- and 12-month-old, and activities of AST and ALT elevated significantly. Meanwhile, incidence of neoplasms was decreased by resveratrol at 9- and 12-month-old, so were activities of AST and ALT. The results at biochemical level were consistent with histological alterations.

Incidence, number, size and degree of spontaneous neoplasms, and activities of serum hepatic marker enzymes increased with age, indicating that *N. guentheri* could represent a promising model for age-dependent tumorigenesis. Resveratrol decreased these parameters and inhibited age-dependent liver spontaneous neoplasms. Next, the underlying mechanism was explored to elucidate that resveratrol inhibited spontaneous liver neoplasms of the new fish model.

### Resveratrol attenuated aging-induced downward trend of SIRT1 in liver neoplasms

SIRT1, a NAD+-dependent histone deacetylase, regulates important biological processes including neuronal protection, organ metabolism, cell apoptosis, cell senescence and tumorigenesis. Early studies identifies that resveratrol activates SIRT1 and inhibits tumor development in SIRT1-dependent manner in cancer cells, nude mice or transgenic mice [23–29]. To determine the role of SIRT1 in spontaneous neoplasms, SIRT1 level at three stages was detected in the fish. In control fish, SIRT1 expressed at a much lower level at 12-month-old than that at 6- and 9-month-old (p<0.05). After long-term resveratrol supplementation, SIRT1 protein was elevated significantly at 6-, 9- and 12-month-old (Figure 1G) (p<0.05). Immunohistochemical staining showed that SIRT1 level decreased in partial neolpasms of control fish, but increased in all of neolpasms in resveratrol-fed fish (Figure 1H). It confirmed that SIRT1 served as a tumor suppressor and resveratrol improved its expression apparently in spontaneous neoplasms of the fish.

### Resveratrol inhibited proliferation and induced apoptosis with up-regulation of SIRT1 in spontaneous neoplasms

#### Resveratrol inhibited proliferation through post-translational modulations of K-Ras mediated by SIRT1

**Figure 2:**
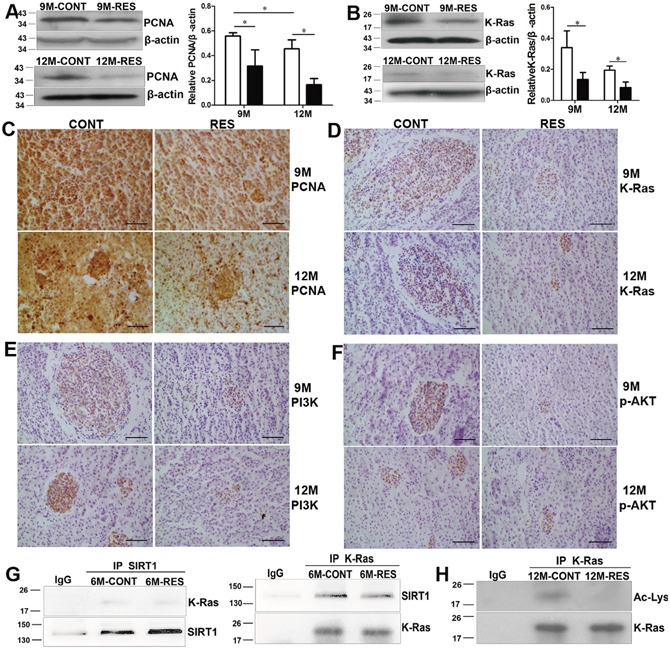
Resveratrol inhibited proliferation by SIRT1-mediated post-translational modulations of K-Ras **(A, B)** Expression of PCNA and K-Ras measured by immunoblot at 9- and 12-month-old (n = 3 in each group). **(C-F)** Immunohistochemical analysis for PCNA, K-Ras, PI3K and p-AKT at 9- and 12-month-old (n = 3 in each group). **(G)** Interaction of K-Ras and SIRT1 at 6-month-old (n = 5 in each group). **(H)** Effects of resveratrol on acetylation of K-Ras (Ac-Lys) at 12-month-old (n = 5 in each group). Scale bar = 50 mm. Data were presented as mean±SD. *p <0.05.

Proliferation marker PCNA was detected in the liver to indentify the effect of resveratrol on cell proliferation. Less PCNA protein was found in resveratrol-fed group than in control group by immunoblot (Figure 2A), and decreased PCNA staining was observed both at neolpasms and pericarcinous tissues in resveratrol-fed fish by immunohistochemistry (Figure 2C), indicating that resveratrol inhibited proliferation of neolpasms.

Overexpression of K-Ras promotes cell proliferation through activation of PI3K/AKT signal in transgenic zebrafish [7]. To identify the role of K-Ras/PI3K/AKT signal in spontaneous neoplasms, protein levels of K-Ras, PI3K and p-AKT were measured. In resveratrol-fed fish, K-Ras level reduced significantly at 9- and 12-month-old (Figure 2B) (p<0.05). The neoplasms showed K-Ras positive immunoreactivity and resveratrol declined K-Ras immunoreactivity (Figure 2D). Likewise, immunostaining showed decreased protein levels of PI3K and p-AKT in hepatic neolpasms of resveratrol-fed groups both at 9- and 12-month-old (Figure 2E, 2F). These results indicated that resveratrol inhibited proliferation was associated with inactivation of K-Ras/PI3K/AKT signaling.

To unravel the mechanistic details that SIRT1 activator resveratrol down-regulated K-Ras/PI3K/AKT pathway, we checked whether there was an interaction between endogenous SIRT1 and K-Ras. Liver proteins were immunoprecipitated with anti-SIRT1 antibody or with control IgG. Immunoblot analysis displayed that K-Ras was detected in the immunoprecipitations obtained with the anti-SIRT1 antibody but not with IgG. A reciprocal co-immunoprecipitation (Co-IP) assay showed similar result, implying that the two proteins interacted with each other in liver of the annual fish (Figure 2G). In cancer cells and transgenic mice, K-Ras deacetylation involves in proliferation of tumor cells [30, 31]. In order to assess whether SIRT1 deacetylated K-Ras in spontaneous neoplasms of the annual fish, we examined acetylation of K-Ras by immunoprecipitating equal amount of K-Ras protein (using few beads incubated with excess total proteins). Result showed that acetylation of K-Ras was decreased by long-term resveratrol feeding (Figure 2H), suggesting that resveratrol blocked K-Ras/PI3K/AKT pathway through deacetylase SIRT1 in spontaneous neoplasms.

#### Resveratrol induced apoptosis through post-translational modulations of FoxOs and DLC1 mediated by SIRT1

**Figure 3:**
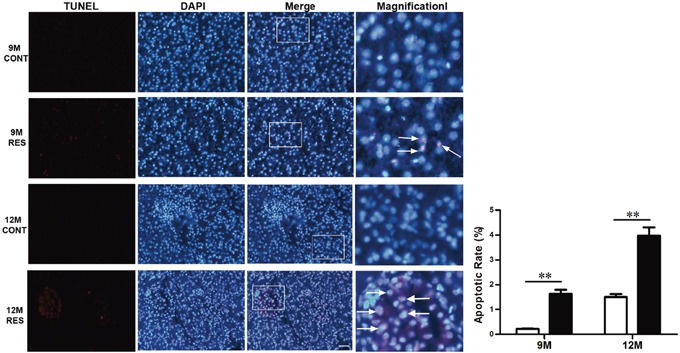
Resveratrol induced apoptosis in spontaneous neoplasms Apoptosis assay was performed in 9- and 12-month-old fish. Scale bar = 10 mm (n = 3 in each group). (Arrowheads) Apoptotic cells.

TUNEL assay was employed to examine cell apoptosis. Apoptotic cells were observed rarely in liver at 6-month-old (data not shown). Liver sections displayed fewer apoptotic cells in control group both at 9-month-old (0.216%±0.016%) and 12-month-old (1.497%±0.118%), and a dramatic increase of apoptotic cells in resveratrol-fed group (1.630%±0.165% at 9-month-old and 3.965%±0.341% at 12-month-old, p<0.01), especially apoptotic foci distributed widely in neoplasms at 12-momth-old (Figure 3). These results indicated that resveratrol significantly induced apoptosis in spontaneous liver neoplasms.

**Figure 4:**
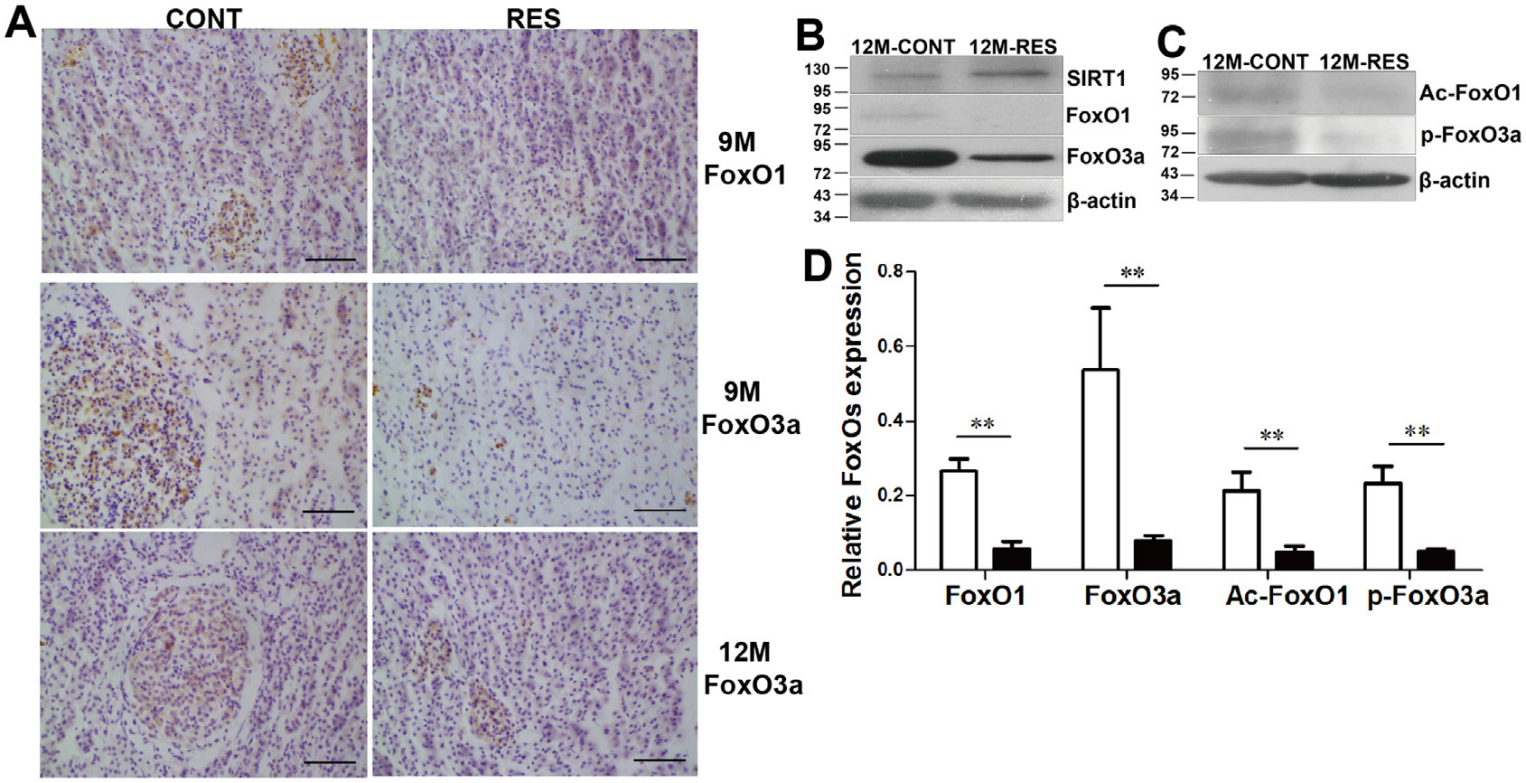
Resveratrol induced apoptosis by down-regulation of FoxO1 and FoxO3a, deacetylation and dephosphorylation of FoxOs with SIRT1 up-regulation **(A)** Immunohistochemical analysis of FoxO1 and FoxO3a at 9- and 12-month-old. **(B, C)** Expression levels of FoxO1, FoxO3a, Ac-FoxO1 and p-FoxO3a at 12-month-old. **(D)** Quantification of FoxOs expression at 12-month-old in control and RES groups. Scale bar = 50 mm (n = 3 in each group). Data were presented as mean±SD. **p <0.01.

SIRT1 deacetylase regulates post-translational modulations of FoxOs, and acetylation and phosphorylation of FoxOs have dual effects on apoptosis by proapoptotic proteins in cancer cells or orthotopic tumors [3, 4, 32]. To explore the mechanisms underlying the proapoptotic effect of resveratrol in spontaneous neoplasms, protein levels and post-translational modulations of FoxOs were assayed. Resveratrol caused a steady reduction of FoxO1 and FoxO3a with increase of SIRT1 at 9- and 12-month-old (p<0.01) (Figure 4A, 4B, 4D). Immunohistochemistry further confirmed that resveratrol reduced FoxO1 and FoxO3a staining in neoplasm foci (Figure 4A). Two post-translational modulations of FoxOs were tested by immunoblot. Results showed that resveratrol negatively regulated expression of Ac-FoxO1 and p-FoxO3a (p<0.01) (Figure 4C, 4D), revealing that resveratrol decreased phosphorylation of FoxO3a (by inactivating K-Ras/PI3K/AKT signal) and acetylation of FoxO1 with SIRT1 up-regulation to induce apoptosis in spontaneous neoplasms.

**Figure 5:**
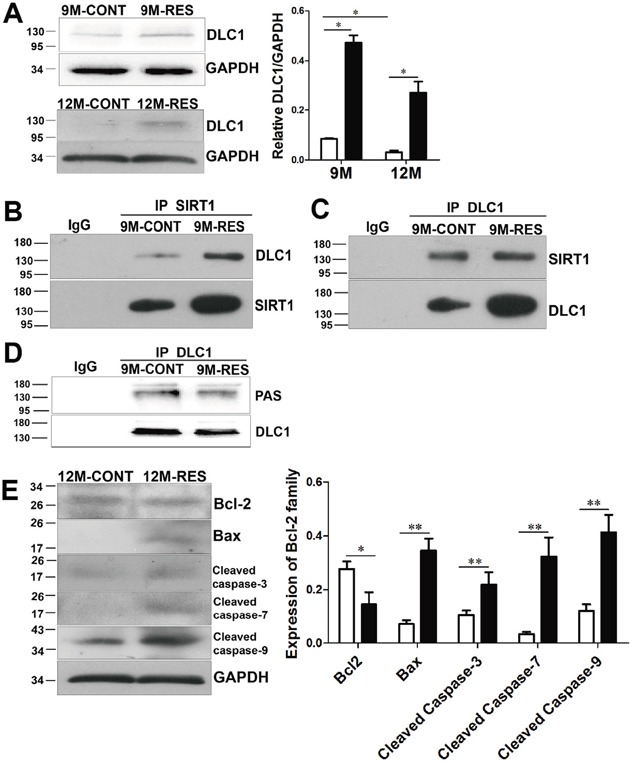
Resveratrol induced apoptosis by up-regulation of DLC1 and interaction between DLC1 and SIRT1, and dephosphorylation of DLC1 with SIRT1 up-regulation **(A)** DLC1 expression at 9- and 12-month-old (n = 3 in each group). **(B, C)** Effects of resveratrol on interaction of DLC1 and SIRT1 at 9-month-old (n = 10 in each group). **(D)** Effects of resveratrol on phosphorylation of DLC1 (PAS) at 9-month-old (n = 5 in each group). **(E)** Levels of Bcl-2, Bax, cleaved capsase-3/7/9 at 12-month-old (n = 3 in each group). Data were presented as mean±SD. *p <0.05, **p <0.01.

DLC1, a tumor suppressor gene, is firstly found in human primary hepatocellular carcinoma [33]. Our previous results show that the full tumor suppressor activity of DLC1 depends on its LD-like motif, which binds talin and FAK, and cooperation between tensin binding and RhoGAP activity [34, 35]. DLC1 exhibits tumor suppressor activity when ectopically expressed in cancer cells [36, 37] and in nude mice injected subcutaneously with lung cancer cells [34, 38]. However, little is known about the function of DLC1 in the progression of spontaneous neoplasms. In the annual fish, our results clearly showed that DLC1 level was markedly lower at 12-month-old than that at 9-month-old of control group, resveratrol elevated DLC1 expression at the two stages (p<0.05). DLC1 expression was still at a lower level although resveratrol increased it in fish at 12-month-old (Figure 5A). So we chose fish at 9-month-old to perform next experiments.

Histone deacetylase inhibitor activates DLC1 promoter in prostate and gastric cancer cells [39–41]. In order to explore the relationship of endogenous SIRT1 and DLC1, Co-IP between deacetylase SIRT1 and DLC1 was performed. Western blot analysis displayed that DLC1 was clearly detected in the immunoprecipitations obtained with the anti-SIRT1 antibody but not with the control IgG (Figure 5B). A reciprocal Co-IP assay was also conducted. As shown in Figure 5C, endogenous SIRT1 was readily immunoprecipitated with DLC1-specific antibody, but not with IgG antibody. And resveratrol enhanced the interaction of SIRT1 and DLC1 in 9-month-old fish.

DLC1 can be phosphorylated (by PKA, PKB, PKC and PKD) and its phosphorylation has positive or negative effect on its tumor suppressor activity in cancer cells and in nude mice [42–45]. In spontaneous neoplasms, we found that resveratrol decreased phosphorylation of DLC1 and up-regulated its tumor suppressor activity *in vivo* (Figure 5D). Overexpression of SIRT1 decreased AKT (PKB) level and further inhibited phosphorylation of DLC1 in spontaneous neoplasms by resveratrol.

Overexpression of DLC1 contributes to apoptosis by up-regulating level of Bax and activity of caspase-3 in hepatocellular carcinoma and cutaneous squamous cell carcinoma cell lines [36, 37]. So we detected downstream proteins of DLC1 and FoxOs to explore mechanism that resveratrol induced apoptosis in spontaneous neoplasms. At 12-month-old, levels of Bax, cleaved caspase-3/7/9 became dramatically higher in resveratrol feeding fish than that in control fish (p<0.01), whereas Bcl-2 displayed the opposite results with above proapoptotic proteins (p<0.05) (Figure 5E). Apoptosis induced by resveratrol might be resulted from deacetylation and dephosphorylation of FoxOs, up-regulation of DLC1 and interaction between SIRT1 and DLC1, and dephosphorylation of DLC1 in spontaneous neoplasms of the fish.

## DISCUSSION

In the present study, we found that hepatic neoplasm was an age-related disease, and resveratrol inhibited age-dependent spontaneous tumorigenesis by SIRT1-mediated post-translational modulations of K-Ras, FoxOs and DLC1 to proliferation and apoptosis in the annual fish.

Di Cicco identifies that *N. furzeri* is an emerging model to study age-dependent spontaneous tumorigenesis as neoplasm incidence increases with age [15]. In the present study, macroscopic dots (histological analysis revealed these dots were neoplasms) were observed by naked eyes in many livers from middle-aged and old fish, and liver weight and relative live weight showed an upward trend with age. Incidence of neoplasms was 86.67% at 9-month-old and it approached 100% in 12-month-old fish. By in-depth analysis, size, number and malignant degree of hepatic neoplasms, activities of serum hepatic marker enzymes increased as aging. These results revealed that *N. guentheri* acted as excellent vertebrate model for spontaneous tumorigenesis. Previous and our present results further imply that fish in genus *Nothobranchius* is a promising model for age-dependent tumorigenesis.

SIRT1 is a NAD+-dependent histone deacetylase that helps to regulate lifespan, oxidative stress and tumorigenesis in diverse organisms [46]. However, the role of SIRT1 during tumorigenesis is currently under debate due to some different findings. SIRT1 level is actually lower in hepatic cell carcinoma, bladder carcinoma and ovarian cancer indicating that SIRT1 may act as a tumor suppressor [28]. Also, SIRT1 is found significantly elevated in human prostate cancer and primary colon cancer [47] implying SIRT1 serves as a tumor promoter. Resveratrol, SIRT1 activator, has multiple biological effects, such as antioxidant activity, anti-inflammatory and anti-cancer [10, 27, 48]. Resveratrol exhibits chemopreventive activity against liver cancer initiated by diethylnitrosamine in rat and induced by orthotopic implant in nude mice [49, 50]. Resveratrol increases expression of SIRT1 in gastric cancer cells and prostate cancer cells [27, 51], and in rats and mice with high-fat diet [52, 53]. Chemopreventive effect of resveratrol is SIRT1-dependent as described that resveratrol does not inhibit growth of tumors in SIRT1 mutant or null mice [28, 29]. However, the role of SIRT1 in the progression of spontaneous neoplasms remains elusive.

SIRT1 involves in protection of antioxidant defense system *in vitro* and *in vivo*. Fullerenol alleviates the depletion of cellular antioxidants and reduces genomic DNA damage induced by H_2_O_2_ via activating SIRT1 in retinal pigment epithelial cells [54]. Resveratrol is able to decrease hepatic steatosis and lipid metabolic disorder, and enhance the antioxidant ability by up-regulating SIRT1 expression in KKAy mice [55]. Melatonin attenuates oxidative stress induced by sepsis via SIRT1 signaling activation in C57BL/6J mice [56]. Moreover, several compounds exhibit anti-cancer effect by decreasing oxidative stress. Combined treatment with curcumin and resveratrol results in a noticeable inhibition of the lung carcinogenesis with elevation of antioxidant activities in mice [57]. Resveratrol inhibits proliferation of cancer cells due to activation of antioxidant enzymes in prostate, hepatic and breast cancer cells [58]. We showed for the first time that SIRT1 expression decreased as aging, and increased with resveratrol feeding at three different ages. Our previous paper shows that oxidative stress increases with age and is attenuated by resveratrol [10]. So we speculated that down-regulation of SIRT1 resulted in oxidative stress and further promoted hepatocarcinogenesis as aging, and resveratrol inhibited spontaneous neoplasms by up-regulation of SIRT1 and decrease of oxidative stress in the annual fish.

Tumor suppressor gene DLC1, firstly isolated from human primary hepatocellular carcinoma, is located on chromosome 8p21-22 and encodes a Rho GTPase-activating protein [33]. DLC1 is expressed widely in normal tissues, but undetectable or underexpressed frequently in various tumors [36, 37, 59], predominantly by heterozygous deletion, promoter hypermethylation and post-translational modification [43]. Overexpression of DLC1 stimulates cell apoptosis by elevating caspase-3 activity and decreasing Bcl-2 level in hepatocellular carcinoma and cutaneous squamous cell carcinoma cells [36, 37]. DLC1 expression can be induced by histone deacetylase inhibitors in prostate and gastric cancer cells [39–41]. Here we firstly found that DLC1 protein level decreased with age from fish at 9- to 12-month-old, and age-dependent increase of spontaneous neoplasms might also caused by down-regulation of the tumor suppressor gene. After long-term feeding, resveratrol elevated DLC1 expression and its interaction with SIRT1, and promoted apoptosis by up-regulating proapoptotic proteins (Bax, cleaved caspase-3/7/9) and further alleviated age-dependent spontaneous tumorgenesis in the annual fish.

Histone deacetylase SIRT1 can lead to activation or inhibition of many proteins such as p53, K-Ras, FoxOs and PGC-1α through post-translational modulations. Acetylation of K-Ras, phosphorylation and acetylation of FoxOs, and phosphorylation of DLC1 have been reported to have dual effects on proliferation and apoptosis in cancer cell lines, orthotopic cancers of nude mice or transgenic mice. But if post-translational modulations of these proteins involve in spontaneous neoplasms remain untested. The K-Ras proto-oncogene is involved in tumorigenesis. In lung caner cells, deacetylation of K-Ras by SIRT1 promotes cell proliferation [30]. In transgenic mice with K-Ras mutant, SIRT2 deletion enhances tumorigenesis by up-regulating acetylation of K-Ras [31]. FoxOs transcription factors play a pivotal role in oxidative stress, genomic damage, cell cycle and apoptosis [60]. FoxO1 acetylation promotes cell apoptosis in pancreatic cancer cells [4]; whereas FoxO3a deacetylation up-regulates expression of proapoptotic proteins in lung cancer cells [32]. FoxOs phosphorylation has dual effects on apoptosis in pancreatic cancer cells [3, 4]. Phosphorylation of DLC1 induced by protein kinases (PKA, PKB, PKC and PKD) regulates biological activity of DLC1. For example, phosphorylation of DLC1 by PKA enhances RhoGAP activity of DLC1, and further suppresses proliferation and metastasis both in hepatoma cells and in nude mice by mouse hepatoma cell subcutaneous injection [42]. DLC1 can be phosphorylated upon expression of AKT or insulin induction in liver cancer cell lines, and S567A mutant (phosphodefective mutant of DLC1) inhibits colony formation in cancer cells and tumor formation in nude mice [43]. PKD also directly phosphorylates DLC1 and negatively regulates DLC1 activity in breast cancer cells [44]. DLC1 phosphorylation by PKC and PKD is required for interaction with 14-3-3 protein and inhibits the RhoGAP activity of DLC1 in breast cancer cells [45]. In the annual fish, resveratrol down-regulated acetylation of K-Ras, phosphorylation of FoxO3a, acetylation of FoxO1 and phosphorylation of DLC1 by SIRT1 or SIRT1-mediated inactivation of K-Ras/PI3K/AKT signal, and inhibited cell proliferation and promoted cell apoptosis in spontaneous neoplasms.

In the annual fish *N. guentheri*, spontaneous hepatic neoplasms were observed at 9-month-old and its incidence approached to 100% at 12-month-old. Age-dependent increase of spontaneous liver neoplasms might result from aging-induced down-regulation of SIRT1 and DLC1. After long-term feeding, resveratrol inhibited age-associated liver spontaneous neoplasms and increased expression of SIRT1 and DLC1 and their interaction. With up-regulation of SIRT1, resveratrol inhibited proliferation (with reduction of K-Ras/PI3K/AKT signal) through post-translational modulations of K-Ras, and induced apoptosis through dephosphorylation of FoxO3a, deacetylation of FoxO1 and dephosphorylation of DLC1. These results demonstrated that resveratrol efficiently inhibited age-dependent spontaneous tumorigenesis by SIRT1-mediated post-translational modulations of K-Ras, FoxOs and DLC1 in the fish. These results highlighted the essential role of SIRT1 in development of spontaneous neoplasms and provided evidence for the annual fishes to act as an excellent vertebrate model for spontaneous tumorigenesis.

## MATERIALS AND METHODS

### Fish and diet

The protocol in this study was approved by the Ethics Committee on Animal Experiments of Medical School of Shandong University (Permit Number: ECAESDUSM 1420123009). The fish *N. guentheri* was bred and reared in our own laboratory. When the fish grew to sexual maturity at 4-month-old, they were randomly separated into control group with standard food and resveratrol feeding group with resveratrol-supplemented food (200 μg/gram food, R5010, Sigma, St. Louis) made as previously [10, 18] until fish were sacrificed at 6-, 9- and 12-month-old. Approximately, the dose was 25 μg resveratrol/fish/day.

Total 350 fish were documented to investigate effects of resveratrol on inhibition of hepatic neoplasms. The exact number of fish in every assay was shown in Supplementary Table 1.

### Relative liver weights

Livers were isolated and the weights were measured immediately. Each liver was observed for gross liver morphology to examine if there were macroscopic dots on the surface. The relative liver weights were expressed as liver weight in gram/100 grams body weight [38]. At least 10 fish were used in each group at 6-, 9- and 12-month-old.

### Histological study

Liver were freshly dissected, fixed in 10% formaldehyde. Sections (7 μm) were stained with hematoxylin and eosin to examine histological changes. Number of fish with nodules and total number of nodules were recorded, nodule incidence and average number of nodules were calculated in each group [22]. We quantified total number of nodules throughout a semi-quantification method. Three rectangular frames for each fish were analyzed randomly [18]. At least 10 fish were used in each group at three stages.

### Activities of hepatic marker enzymes

Blood was collected from caudal vein and clotted for 2 hours at room temperature, and serum was separated by centrifugation at 3000g for 15 min [61]. Activities of AST and ALT in serum were detected according to the manufacturer's instructions of analysis kits (Nanjing jiancheng, China). At least 6 fish were used to in each group at three stages.

### Immunohistochemical analysis

The sections were boiled for antigen retrieval in 10 mM citrate buffer pH 6.0 for 10 min after rehydration, treated with 3% hydrogen peroxide for 15 min, and blocked using 5% BSA for 30 min. Then, the sections were incubated with primary antibody against SIRT1, PI3K, FoxO3a and p-AKT (Cell Signaling Technology, Massachusetts); mutant p53, PCNA and K-Ras (Santa Cruz Biotechnology, Dallas); and FoxO1 (Bioworlde, China) overnight at 4°C and incubated in corresponding secondary antibody for 1 hour at room temperature. Lastly, signals were detected using DAB substrate kit (ZLI-9018, ZSGB-BIO, China). At least 3 fish at each age in each group were used for every marker measured by immunohistochemical analysis.

DNA strand breaks generated during apoptosis were stained using *in situ* Cell Death Detection Kit (TMR red) according to the manufacturer's instructions (12156792910, Roche, Switzerland). At least 3 fish both in control and resveratrol-fed group at three stages were used to detect cell apoptosis.

### Western blot analysis

Total proteins were exacted from liver samples using lysis buffer (P0013B, Beyotime, China) containing 1mmol/L PMSF. Protein concentration was determined by BCA protein assay kit (PA115, TIANGEN, China). 120μg of proteins were separated in 10% or 12% SDS-PAGE and transferred to PVDF membranes. After blocking with 5% milk, membranes were incubated with primary antibody against SIRT1, K-Ras, PCNA, FoxO3a, FoxO1, Ac-FoxO1 (Santa Cruz Biotechnology), Bcl-2 (Proteintech, China), DLC1 (BD Biosciences, Franklin Lakes, USA), p-FoxO3a, Bax, cleaved caspase-3/7/9 (Cell Signaling Technology), GAPDH (Affinity, China) and b-actin (ZSGB-BIO, China) overnight at 4°C. Then the membranes were incubated in secondary antibody for 90 min. Protein expression was detected with the ECL system (P0018, Beyotime, China). At least 3 fish at each age in each group were used for every marker measured by immunoblot.

Immunoprecipitation was used to investigate interaction between SIRT1 and K-Ras, acetylation of K-Ras, interaction of SIRT1 and DLC1, and phosphorylation of DLC1. Total proteins of 500-1000 μg were incubated with 2 μg SIRT1 (Santa Cruz Biotechnology), K-Ras or DLC1 antibodies for 2 h for immunoprecipitation. Immune complexes were further incubated with 25 μl (for interaction of two proteins) or 10 μl (for acetylation of K-Ras and phosphorylation of DLC1) protein A/G agarose beads (88804, Thermo scientific, Massachusetts) for 1.5 h. 100 μg proteins were resolved by SDS–PAGE and detected with SIRT1, K-Ras, Ac-Lys, DLC1 or PAS (Cell Signaling Technology) antibodies as before indicated. At least 5 fish at each stage in each group were used for immunoprecipitation.

The catalog and dilution of antibodies used in immunohistochemical, western blot and immunoprecipitation were depicted in Supplementary Table 2.

### Statistical analysis

All of the assays were repeated at least in three fish independently. Figures were processed by Graphpad Prism 5 and Adobe Illustrator CS6. Statistical analysis was performed using the SPSS 17.0 software. Data were presented as mean±standard deviation (SD). Differences were analyzed by one way ANOVA. P-values of <0.05 were considered statistically significant.

## SUPPLEMENTARY MATERIALS FIGURES AND TABLES



## Abbreviations

SIRT1: sirtuin 1
DLC1: deleted in liver cancer-1
RES: resveratrol
AST: aspartate aminotransferase
ALT: alanine transaminase
Co-IP: co-immunoprecipitation
PAS: phospho-AKT substrate.
