# Supplementary material for: Resveratrol inhibits age-dependent spontaneous tumorigenesis by SIRT1-mediated post-translational modulations in the annual fish Nothobranchius guentheri
Source: Oncotarget. 2017 Jul 15;8(33):55422–34. doi: 10.18632/oncotarget.19268 (PMC5589669; doi:10.18632/oncotarget.19268)
Supplement: Supplementary file 1 [file oncotarget-08-55422-s001.pdf]

Resveratrol inhibits age-dependent spontaneous tumorigenesis by SIRT1-mediated post-translational modulations in the annual fish *Nothobranchius guentheri*

SUPPLEMENTARY MATERIALS

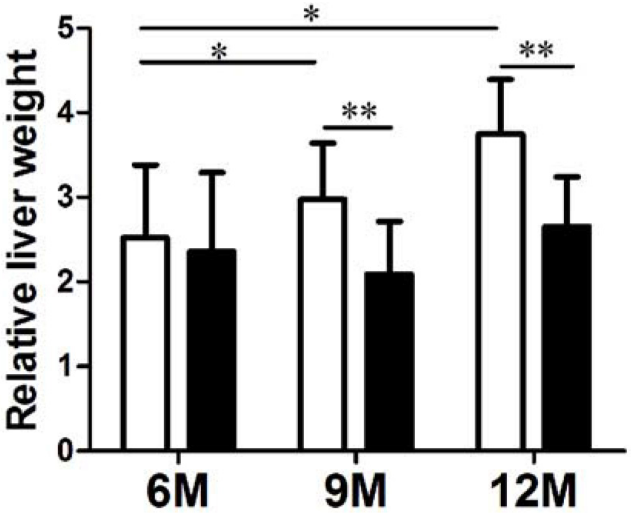

Supplementary Figure 1: The relative live weight of the annual fish at 6-, 9- and 12-month-old. Resveratrol decreased relative live weight significantly at last two stages.

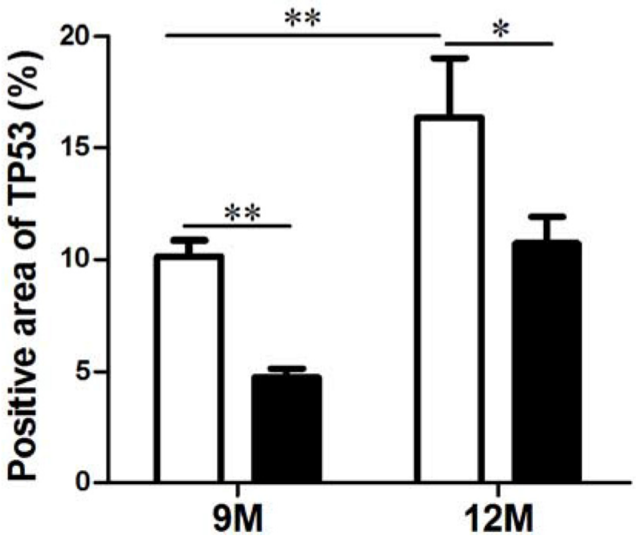

Supplementary Figure 2: The level of TP53 immunoreactivity at 9- and 12-month-old. Resveratrol reduced positive staining of TP53 at the two stages.

Supplementary Table 1: Description of number of fish used for each experimental approach

Assay	6M CONT	6M RES	9M CONT	9M RES	12M CONT	12M RES
Relative liver weights and Histological study	10	10	10	10	10	10
AST, ALT	6	6	6	6	6	6
SIRT1	3	3	6	6	6	6
PCNA, TP53			6	6	6	6
K-Ras			6	6	6	6
PI3K, p-Akt			6	6	6	6
FoxO1, Bcl-2			3	3	3	3
FoxO3a, Bax			3	3	6	6
Ac-FoxO1, cleaved caspase-3					6	6
p-FoxO3a cleaved caspase-7					6	6
DLC1 cleaved caspase-9			3	3	6	6
Tunel	5	5	5	5	5	5
IP: SIRT1 and K-Ras	5	5				
IP: ac-KRas					5	5
IP: SIRT1 and DLC1			10	10		
IP: PAS			5	5		

Supplementary Table 2: Antibodies used in this study along with dilutions

Antibody	Catalog	Molecular wt	Assay	Dilution
SIRT1	CST (8469)	120 KD	WB/IMC	1:1000/1:200
TP53	Santa(sc-99)		IMC	1:200
PCNA	Santa (sc-7907)	36 KD	WB/IMC	1:1000/1:200
K-Ras	Santa (sc-30)	21 KD	WB/IMC	1:1000/1:200
PI3K	CST (4249)		IMC	1:200
p-AKT	CST (4060)		IMC	1:200
FoxO1	Bioworld (BS1746)	70-82 KD	WB/IMC	1:1000/1:200
FoxO3a	CST (12829)	82-97	WB/IMC	1:1000/1:200
Ac-FoxO1	Santa (sc-49437)	70	WB	1:1000
p-FoxO3a	CST (9465)	97	WB	1:1000
DLC1	BD (612021)	123	WB/IP	1:1000/ 0.5-1mg protein: 2ug antibody
SIRT1	Santa (sc-74465)	120	IP	0.5-1mg protein: 2ug antibody
Ac-Lys	CST (9441)		IB	1:1000
Bcl-2	Proteintech (12789-1-AP)	26	WB	1:1000
Bax	CST (2772)	20	WB	1:1000
Cleaved caspase-3	CST (9664)	17, 19	WB	1:1000
Cleaved caspase-7	CST (8438)	18	WB	1:1000
Cleaved caspase-9	CST (7237)	37	WB	1:1000
Actin	Proteintech (66009-1-lg)	42	WB	1:2000
GAPDH	Affinity (T0004)	34	WB	1:2000
PAS	CST(9611S)		IB	1:1000

CST: Cell Signaling Technology.

Santa: Santa Cruz Biotechnology.

BD: BD Biosciences.
